# Evaluation of the Responsible, Engaged, and Loving (REAL) Fathers Initiative on Physical Child Punishment and Intimate Partner Violence in Northern Uganda

**DOI:** 10.1007/s11121-016-0713-9

**Published:** 2016-10-13

**Authors:** Kim Ashburn, Brad Kerner, Dickens Ojamuge, Rebecka Lundgren

**Affiliations:** 1 Institute for Reproductive Health, Georgetown University, 1825 Connecticut Avenue, NW, Suite 699, Washington, DC 20009 USA; 2 Save the Children US, 501 Kings Highway, Fairfield, CT 06825 USA; 3 Save the Children International, Plot 1 Arthur Oryem Road, PO Box 353, Gulu, Uganda

**Keywords:** Uganda, Intimate partner violence, Child maltreatment, Men, Gender

## Abstract

Violence against women and violence against children in Uganda are recognized as significant public health concerns. Exposure to violence at home as a child can increase the likelihood of perpetrating or experiencing violence later in life. These two forms of violence share similar risk factors and often, but not always, co-occur at the household level. Parenting programs have shown promise in reducing physical child punishment. Targeting men has also been proven effective in transforming attitudes related to gender roles and expectations and intimate partner violence (IPV) against women. The REAL Fathers Initiative is a 12-session father mentoring program implemented by volunteers that is designed to reduce child exposure to violence at home, breaking the cycle of intergenerational violence. Evaluation results comparing survey data among men exposed to the intervention and those unexposed demonstrate significant reductions in IPV at end line (aOR 0.48, CI 0.31, 0.76, *p* < 0.001) and over the longer term follow-up (aOR 0.47, CI 0.31, 0.77, *p* < 0.001) and significant reductions in physical child punishment at long-term follow-up (aOR 0.52, CI 0.32, 0.82, *p* < 0.001).

## Introduction

Violence against women and violence against children exact a serious toll on the health and well-being of families across Uganda. According to population survey data, 60 % of Ugandan women in union reported ever experiencing intimate partner violence (IPV)—physical, emotional, or sexual violence—by their current partner or spouse, and 45 % reported experiencing IPV in the past year (Uganda Bureau of Statistics 2012). Prevalence of violence against children or child maltreatment (CM) as defined by WHO (includes “all forms of physical and emotional ill treatment, sexual abuse, neglect, and exploitation”) is even higher. According to results of a survey among 1000 children aged 8 to 18 years across five districts (covering a range of cultures and levels of urbanization), more than 98 % of children reported experiencing physical or emotional violence and 76 % sexual violence (Naker 2005). Health consequences of both IPV and CM include not only the obvious physical injuries but also a range of negative consequences including social, developmental, and economic. In Uganda, women who have experienced IPV are more likely to have depression, problem drinking, and attempted suicide (Kinyanda et al. 2016); HIV infection (Wagman et al. 2015); and physical injury (Koenig et al. 2003).

Several multicountry studies, including East African countries, provide evidence that these two forms of violence may occur across generations (Fleming et al. 2015; Hindin et al. 2008) and that they may share similar risk factors such as gender inequality (Guedes et al. 2016). Several studies from Uganda indicate a link between witnessing IPV at home as a child and increased risk of perpetrating or experiencing IPV as an adult in both men and women (Speizer 2010), and for women, increased odds of experiencing intimate partner physical violence (Kwagala et al. 2013). High levels of IPV and CM in post-conflict Northern and Karamoja regions may be due to previous exposure to sexual and physical violence during the conflict, which is now resulting in perpetration and victimization through an intergenerational cycle of violence (Saile et al. 2014). Kinyanda et al. (2016) observed high rates of physical and sexual violence victimization among women who were exposed to sexual and physical violence during the armed conflict in Eastern Uganda.

Parenting programs have shown promise in curbing CM. Mikton and Butchart (2009) reviewed systematic reviews of interventions designed to prevent CM. The authors found that most research on prevention of CM was conducted in high-income countries and less than 1 % of the studies identified were in low-income or middle-income countries. Of the seven types of interventions reviewed, four were determined to be promising including parent education interventions, which addressed CM through improving parenting skills, increasing parents’ knowledge of child development, and promoting positive behavior management strategies. Parenting programs addressing CM in post-conflict settings provide rigorous evidence of declines in CM in Liberia (Sim et al. 2014b) and Thailand (Sim et al. 2014a). Programs targeting fathers as caregivers have generally focused on CM, with a few exceptions such as Program P in Rwanda, which addressed IPV and CM (Doyle et al. 2014). Analysis of qualitative and quantitative data from SASA!, a community-based HIV and IPV intervention in Uganda, show important unintended effects on improved parenting, including improved parent-child relationships and less frequent use of physical punishment (Kyegombe et al. 2015). Given that exposure to IPV during childhood can be a risk factor for CM, and experience of violence later in life, there is increasing interest in exploring the integration of CM and IPV interventions.

Social and gender norms influence the acceptance and occurrence of IPV (Heise 2011). In a randomized controlled trial (RCT) in rural South Africa, the combination of community mobilization, a training curriculum, Sisters for Life, and access to microcredit, reduced IPV by 55 % (Pronyk et al. 2006). Two RCTs of community-based interventions addressing gender inequality and IPV provide evidence of reductions in IPV. Exposure to Stepping Stones in South Africa led to a decrease in the acceptability of IPV among women (Jewkes et al. 2008). Evaluation results of SASA! in Uganda included decreases in physical, sexual, and emotional IPV against women (Abramsky et al. 2014). In a review of IPV prevention programs, Lundgren and Amin (2015) found limited evidence of effectiveness of interventions that focus on women only. Several studies have shown that working with men can have positive effects on improving attitudes related to gender inequality and in reducing perpetration of IPV (Jewkes et al. 2008).

Few interventions were identified in the literature targeting fathers and addressing both CM and IPV, particularly in developing country settings. The REAL Fathers Initiative was designed to address gender norms that promote use of violence in child discipline and with intimate partners through promotion of positive parenting and partnership skills building. This paper describes evaluation results of the REAL Fathers Initiative on key outcomes including attitudes toward use of physical punishment and IPV, confidence in using nonviolent discipline strategies, couple communication, and use of physical punishment and IPV.

## Methods

### Study Site and Study Population

The study was conducted in Attiak sub-county, Amuru district in the Northern region of Uganda. This district was heavily affected by the 20-year war waged by the Lord’s Resistance Army (LRA) against Uganda’s national armed forces. At the height of the conflict, nearly two million people were relocated to internally displaced person (IDP) camps. A high level of violence was documented in these camps during the conflict (Gelsdorf et al. 2012) and has likely contributed to the existing levels of violence in the region (Saile et al. 2014). After the ceasefire, brokered during 2006 to 2008, families began to leave the IDP camps and move back to their traditional villages, reclaiming their land, and rebuilding their homes. The war deeply affected social relationships, leaving younger generations without the support of parents or elders to guide and advise them in important roles in family and community. Traditionally, land was handed down from father to son, but with an increasing scarcity of land, families are pressured to seek income generation opportunities where few are available. With the advent of basic education reforms and mandatory primary and now secondary schooling, parents and caregivers have the added burden of paying school fees. Now, in a period of post-conflict development, the government has been rebuilding schools and strengthening services in the region.

### Participants

Participants were 500 young fathers aged 16 to 25 who have toddler-aged children (1–3 years) and are married or cohabitating with their wife or partner. They were recruited from villages across all eight parishes of Attiak sub-county, an area where the implementing partner, Save the Children International (SCI), previously worked and was well known as a reputable organization. Within the sub-county, Local Council 1 (LC1s), the lowest level administrative elected leader, participated in generating a list of all eligible young fathers from selected villages based on eligibility criteria. The study was conducted in two cohorts. A total of 340 men were recruited into cohort 1 in July 2013 and 160 men in November 2013. Power calculations suggested that a sample size of 500 was sufficient to detect a 5 % reduction in the use of physical violence against their wife/partner between baseline and end line.

### Measures

Measures included in the survey instrument were based on the content of the curriculum and constructs in the literature that influence IPV or physical punishment. Perpetration of IPV and physical punishment measures were adapted from the Conflict Tactics Scale (Strauss et al. 1996) and Parent-Child Conflict Tactics Scale (Straus et al. 1998), respectively. Selection of items was based on those behaviors that are reported most frequently in the Demographic and Health Surveys (DHS) domestic violence module in Uganda and include physical, psychological, and verbal forms of violence. Sexual violence was omitted from the survey because it was not covered in any depth in the curriculum.

IPV: Men were asked, “In the past 3 months have you…?” for each of eight possible actions including “shouted or yelled at your wife?,” “slapped your wife,” and “pushed or shoved your wife,” with possible response options never, sometimes, often, or no response (alpha = 0.76).

Physical punishment: Men were asked, “In the past month, have you done the following to discipline the child…?” for each of seven possible actions including “shook him/her”; “shouted, yelled, or screamed at him/her”; and “spanked, hit, or slapped him/her on the bottom with bare hand,” with response options yes, no, or do not remember (alpha = 0.73).

Independent variables cover constructs known in the literature to predict IPV and physical punishment. Justification for using IPV measures were adapted from the DHS domestic violence module used in Uganda.

Justification for IPV: Men were asked, “In your opinion, is a husband justified in hitting or beating his wife if she…?” for each of three items including “goes out without telling him,” “neglects the children,” or “argues with him” (alpha = 0.66).

Justification for physical punishment: Men were read five statements including “stubborn children need to be hit to teach them right and wrong”; “if a child is old enough to defy a parent, then he/she is old enough to be hit”; and “if you love children, you will hit them when they misbehave” (alpha = 0.79). Response options were agree, partially agree, or disagree.

Two parenting practice measures were developed by the study team and based on constructs in parenting literature such as warmth and involvement that are correlated with child behavior and discipline strategies (Stormshak et al. 2000). These include positive parenting and spending time with the child.

Positive parenting: Men were asked, “In the past month, if the child did something you liked, did you…?” for each of six potential responses including “say something nice about it or praise the child” and “give the child physical affection, e.g., pat on the back” (alpha = 0.77).

Spending time with the child: Men were asked, “In the past 3 days, did you or anyone in the household over 15 years of age engage in any of the following activities with the child…?” for each of six items including “read books or look at books,” “tell stories,” and “sing songs” (alpha = 0.81).

Couple communication measures were developed by the study team. Men were asked, “In the past month, did you…?” for each of five items including “tell your wife you appreciated her” and “take time to listen to your wife” (alpha = 0.77).

All measures are additive indices with binary outcomes using cutoffs at the mean. A single item was developed by the study team to measure confidence in using nonviolent discipline, and several items were adapted from the Gender Equitable Men Scale (Pulerwitz and Barker 2008) to measure attitudes about women’s and men’s roles in child care and decision making. Program exposure was measured as attendance of at least one individual and one group mentoring session.

### Intervention

The REAL Fathers Initiative uses a mentoring program and a community poster campaign. These intervention components are grounded in social cognitive theory, which posits that gender differentiation is a social phenomenon with both psychological and social-structural influences (Bussey and Bandura 1999). Individuals are able to differentiate gender by the early ages of 2 to 4 years. Gender knowledge is one aspect of gender differentiation, but social cognitive theory proposes that individuals self-regulate, adapting their behaviors to adhere to gender roles and expectations across a range of social experiences and contexts. Central to social cognitive theory is learning by observation through modeling of behaviors that allow individuals to expand their knowledge and skills. The REAL Fathers Initiative uses modeling of alternative strategies for nonviolent discipline and conflict resolution to improve fathers’ parenting and communication skills and confidence in adapting nonviolent strategies. Promoting these strategies leads to reductions in the use of either form of violence at home, thus reducing child exposure to IPV and maltreatment over time. Social learning theory takes a life course perspective such that gender conceptions and roles are not static but change across the life span. Intervening with young fathers who are learning new roles as parents and partners is an ideal time to promote nonviolence in parenting and partner relationships as there is still ambiguity in the normative expectations about these roles and behaviors. The theory of change is based on the assumptions that increased knowledge and skills in positive parenting and exposure to alterative nonviolent discipline strategies leads to fathers practicing more positive parenting and improved parent-child interaction. Targeting couple communication skills, joint problem solving, and nonviolent responses to couple conflict reduces perpetration of IPV in the long term. Self-reflection on gender roles, by husbands and wives, and at the community level through exposure to posters, leads to improvement in acceptance of an expanded role for the father over time.

A total of 64 mentors were recruited, each guiding up to four mentees. Each mentor/mentee pair met twice a month for 6 months, once in an individual session and once in a group session of three or four mentors and their mentees. Two of the individual sessions and one group session included the young father’s wife or partner. Each session followed a standard format and took approximately 40 to 90 min. During each session, mentors gave the men assignments to practice new skills. Men’s experiences in completing their assignments and practicing the new skills were discussed during the following session. The curriculum used a “yellow card” strategy adapted from soccer’s yellow warning card system. Designed to avoid the escalation of disagreements into physical violence, the yellow cards could be used by either partner to communicate to their partner that an important issue needed to be discussed or to pause a discussion that has the potential to turn violent. In addition to mentoring sessions, a series of six posters were displayed on sign boards at locations in the community frequented by the young fathers. Posters changed monthly and corresponded with the themes and messages presented during the mentoring sessions. Posters included a photograph of a local father performing one of the desired behaviors, such as reading to his child, and a statement indicating that others approve of that action. After the final mentoring session, an open community meeting, or “community celebration,” took place in each study community and were attended by LC1s, program participants, and their wives and families. These celebrations supported norm change at the community level by providing fathers a public forum to commit to continue practicing new skills and for the LC1s and family members to commit their support for the men’s adoption of positive change.

### Procedures

Mentors were trained in delivering a structured curriculum covering three key themes of fatherhood, parenting, and couple communication. Before the intervention began, mentors received a 5-day training covering the content of the curriculum and values clarification exercises on violence and gender. A 2-day mentor training was held midway through implementation of the curriculum to assess the mentoring and to prepare mentors for the remaining sessions. Mentors were volunteers from the community selected by the young fathers. The selected mentors were interviewed by SCI staff to provide information about the project, ensure their willingness and motivation to participate as a mentor, and to map the location of the mentor's residence relative to the mentee. Mapping the residences of the mentor/ mentee pairs helped facilitate the mentoring visits and in some cases young fathers were asked to select a different mentor who was geographically closer. A meeting was organized by SCI with LC1s to introduce them to the study and solicit their support in identifying eligible young men in their communities. Bicycles were provided to the mentors in recognition of the value of their work and to facilitate meeting regularly with their mentees. Incentives were not provided to mentors or participants.

Effectiveness of the REAL Fathers Initiative was evaluated by comparing men who participated in at least one individual and one group mentoring session (exposed) versus men who did not (unexposed), in two independent samples at end line and at long-term follow-up by using cross-sectional data. Originally designed as an RCT, men were assigned to control and intervention groups by using a lottery method. Unique identification codes were not used due to concerns about confidentiality and the sensitive nature of the research topic. Therefore, the study team was not able to track group assignment. For purposes of this evaluation, men who attended at least one individual and one group mentoring session were included in the exposed group, even if originally assigned to the control group.

Men were surveyed at baseline prior to implementation of the intervention and again after a 10-month intervention period, including a 6-month period of mentoring, posters, and community celebrations and a 4-month post-implementation period. All 500 men were surveyed at long-term follow-up in July 2015, 12 and 8 months for cohorts 1 and 2, respectively, after the end of the intervention period.

The study received ethical approval from institutional review boards at Georgetown University and the Uganda National Council for Science and Technology.

## Results

### Analysis Plan

Bivariate and multivariate tests of association using logistic regression, adjusting for demographic characteristics, including employment, exposure to violence as a child, alcohol use, and couple communication, were calculated to evaluate the effectiveness of the intervention on key parenting and partner relationship outcomes. Sensitivity analysis was conducted by including the 12 men that were most similar to intention to treat for the group randomized to intervention at baseline. These 12 men were exposed to one individual but no group mentoring sessions and were most likely allocated to intervention and dropped out. It cannot be guaranteed that these 12 men were assigned to the intervention group at baseline, and we still found our results robust.

Response rates were high for both end line and longer term follow-up surveys, 87 and 80 %, respectively. Total samples after data cleaning included 500 men at baseline, 435 at end line, and 399 at longer term follow-up. For this paper, men who attended at least one individual and one group mentoring session are compared to men who did not attend any mentoring sessions.

At baseline, 90 % of men had been living together with their partners for at least a year and 99.6 % were biological fathers to the toddler-aged child of focus for the intervention. Nearly all, 98 %, had attended school, but only 36 % had ever attended secondary school. Thse majority of men, 90 %, belonged to the Acholi ethnic group, and 90 % were engaged in farming. Only 10 % of men were employed in the formal or informal sector. A high proportion, 72 %, of all men saw their mother or another woman in their home being beaten during their childhood or were spanked or threatened with physical punishment. In the 1 month prior to the baseline survey, 43 % of men had used physical punishment with the child. A total of 30 % of men felt that a husband was justified in beating his wife for any reason. In the 3 months preceding the baseline, men reported yelling (35 %), pushing (20 %), or slapping (19 %) their wife, respectively.

At end line, a total of 256 (51 %) men had been exposed to at least one individual and one group mentoring session. Of those who were exposed to mentoring, 10 (4 %) men said their wife/partner did not have any conversations with the mentor. Of the entire sample, 66 (13 %) men did not see posters with messages about fatherhood in their community. No significant differences were observed in background characteristics at end line between exposed and unexposed men (Table 1), except for being threatened with physical violence as a child. Significantly more exposed versus unexposed men reported being threatened with violence as a child (*p* value <0.05).

**Table 1:** Demographic characteristics of exposed versus unexposed men at end line (*n* = 435)

	Exposed (*n* = 256) % (*n*)	Unexposed (*n* = 179) % (*n*)	*p* value
Age (mean years)	23	23	0.085
Education			
Lower primary	34 (86)	31 (56)	0.613
Upper primary or higher	66 (170)	69 (123)	
Employment			
Farming	89 (227)	88 (158)	0.825
Self-employed	4 (11)	6 (10)	
Work for employer	5 (12)	3 (6)	
Work for family	4 (9)	2 (5)	
Payment of bride price			
No consent of family/agree to bride price	34 (86)	31 (56)	0.821
Partially or completely paid	66 (170)	69 (123)	
Childhood experiences			
Witnessed violence at home as a child (often or sometimes)	74 (188)	72 (128)	0.627
Threatened with physical punishment as a child (often or sometimes)	80 (202)	70 (124)	0.019
Experienced physical violence as a child (often or sometimes)	91 (231)	86 (153)	0.140
Parented by father	59 (149)	52 (93)	0.186
Use of a family planning method	43 (111)	37 (67)	0.216
Wife’s age (mean years) Among men who know wife’s age (*n* = 171)	21	20	0.169
Wife’s education			
Lower primary	16 (38)	19 (32)	0.432
Upper primary and higher	84 (201)	81 (137)	
Never attended school	6 (15)	8 (20)	
Child’s age (mean years)	3	3	0.560
Child’s sex (girl)	47 (120)	53 (95)	0.203
Biological father of child	99 (254)	98 (176)	0.389
Who makes decisions about money at home			
Me	13 (32)	13 (24)	0.783
My wife	3 (8)	4 (7)	
Both of us	84 (214)	81 (145)

**Table 2:** Demographic characteristics of men at long-term follow-up (*n* = 399)

	Exposed (*n* = 232) % (*n*)	Unexposed (*n* = 169) % (*n*)	*p* value
Age (mean years)	24	24	0.526
Education			
Lower primary	29 (68)	22 (36)	0.093
Upper primary and higher	71 (165)	78 (130)	
Employment			
Farming	92 (214)	88 (146)	0.316
Self-employed	3 (8)	5 (8)	
Work for employer	4 (10)	6 (10)	
Work for family	1 (1)	1 (2)	
Payment of bride price			
No consent/knowledge of family	29 (68)	22 (36)	0.093
Partially or completely paid	71 (165)	78 (130)	
Childhood experiences			
Witnessed violence at home as a child (often or sometimes)	73 (168)	76 (126)	0.476
Threatened with physical punishment as a child (often or sometimes)	73 (168)	76 (126)	0.435
Experienced physical violence as a child (often or sometimes)	83 (193)	90 (149)	0.051
Parented by father	64 (149)	58 (97)	0.264
Use of a family planning method	27 (64)	28 (46)	0.957
Wife’s age (mean years)	21	21	0.451
Wife’s education			
Lower primary	15 (33)	18 (29)	0.429
Upper primary and higher	85 (183)	82 (129)	
Child’s age (mean years)	3	3	0.892
Child’s sex (girl child)	45 (106)	51 (85)	0.260
Biological father of child	99 (231)	100 (166)	0.231
Who makes decisions about money at home			
Me	8 (19)	13 (21)	0.190
My wife	3 (6)	4 (7)	
Both of us	89 (208)	82 (136)

**Table 3:** Parenting and partner relationship outcomes at baseline, end line, and long-term follow-up (LTFU)^a^

Variable	Baseline (*n* = 500)	End line (*n* = 435)	LTFU (*n* = 399)
Alcohol use (in past month) Never/no drinking Drinking, but no more than 2 days Drinking, on more than 2 days	40 (202) 31.8 (159) 27.8 (139)	38.4 (166) 39.1 (169) 22.4 (97)	44.6 (178) 29.8 (119) 25.5 (102)
Parenting			
Positive parenting (uses at least five of six behaviors)	58.6 (293)	74.2 (323)	78.9 (315)
Parent-child interaction (any interaction)	85.4 (427)	90.5 (494)	88.4 (353)
Attitude rejecting physical discipline	43.2 (216)	60 (261)	55.8 (223)
Very confident in using nonviolence discipline	25 (125)	23.2 (101)	35.8 (143)
Physical punishment (past month)	68.6 (343)	54.9 (239)	50.3 (201)
Intimate partner relationship			
Couple communication (uses all four behaviors)	Not measured	72.4 (311)	68.5 (272)
Justification of IPV (justified for any reason)	51.8 (255)	32.7 (138)	27.1 (106)
Any form of IPV (past 3 months)	66.2 (333)	59.4 (259)	36.5 (146)
Physical violence (past 3 months)	37.8 (189)	31.4 (137)	12.2 (49)
Psychological violence (past 3 months)	41.2 (206)	29.8 (130)	17.5 (70)
Verbal violence (past 3 months)	50.8 (254)	48.9 (213)	29.8 (119)

^a^All measures are additive indices with binary outcomes and cutoffs at the mean at baseline, or discrete measures

**Table 4:** Adjusted odds ratios (and 95 % confidence intervals) from multiple logistic regression analysis of associations between program exposure and positive parenting, parent-child interaction, attitudes toward physical punishment, confidence in using nonviolent discipline, and physical punishment at end line (*n* = 435) and long-term follow-up (LTFU) (*n* = 399)

	Positive parenting		Parent-child interaction		Rejects use of physical discipline		Confident in using nonviolent discipline		Physical punishment	
	End line	LTFU	End line	LTFU	End line	LTFU	End line	LTFU	End line	LTFU
Attended at least one individual and one group mentoring session (ref did not attend)	1.6 (1.01, 2.63)*	1.1 (0.65, 1.96)	2.1 (1.05, 4.33)*	2.9 (1.48, 6.01)**	1.6 (1.09, 2.49)**	2.2 (1.43, 3.47)***	2.5 (1.50, 4.28)***	2.4 (1.55, 3.98)***	0.84 (0.55, 1.28)	0.52 (0.32, 0.82)**
Employment (ref farming) Works for employer Self-employed Does not work/other	2.2 (0.85, 5.81)	3.1 (0.87, 1.38)	0.5 (0.19, 1.55)	1.5 (0.44, 5.61)	1.1 (0.57, 2.42)	0.8 (0.88, 1.78)	1.17 (0.53, 2.59)	1.7 (0.83, 3.84)	0.96 (0.47, 3.00)	1.4. (0.66, 3.18)
Partial/full payment of bride price (ref no payment)	0.9 (0.56, 1.52)	1.1 (0.65, 2.19)	0.8 (0.37, 1.69)	1.0 (0.48, 2.31)	1.2 (0.81, 1.88)	0.45 (0.26, 0.76)**	0.82 (0.51, 1.34)	0.90 (0.55, 1.49)	1.9 (1.29, 3.00)**	5.6 (3.23, 9.82)***
Couple communication (ref none)	3.2 (1.96, 5.23)***	2.5 (1.43, 4.50)***	2.0 (1.01, 4.23)*	1.0 (0.67, 2.74)	1.3 (0.84, 2.09)	1.7 (1.07, 2.75)*	0.9 (0.56, 1.65)	2.2 (1.34, 3.80)**	0.6 (0.42, 1.07)	0.6 (0.40, 1.08)
Alcohol use in past month (ref never/no drinking) 2 days or less More than 2 days	0.5 (0.34, 1.01) 0.8 (0.42, 1.51)	0.5 (0.27, 1.01) 0.4 (0.24, 0.91)*	1.1 (0.53, 2.55) 1.3 (0.54, 3.45)	0.6, (0.28, 1.33) 0.8 (0.38, 1.95)	1.3 (0.87, 2.17) 1.2 (0.74, 2.15)	1.0 (0.62, 1.76) 0.4 (0.24, 0.72)**	0.8 (0.52, 1.51) 1.0 (0.58, 1.99)	1.5 (0.91, 2.51) 0.6 (0.38, 1.22)	0.9 (0.60, 1.49) 1.0 (0.60, 1.75)	0.5 (0.35, 1.02) 1.0 (0.62, 1.87)
Threatened with physical violence often/sometimes (ref never)	1.9 (1.15, 3.44)*	6.4 (3.34, 12.51)***	1.0 (0.49, 2.43)	0.8 (0.33, 2.16)	0.67 (0.40, 1.10)	0.4 (0.26, 0.92)*	0.67 (0.38, 1.16)	1.0 (0.57, 1.95)	1.8 (1.12, 2.97)**	2.0 (1.09, 3.78)*
Experienced physical punishment often/sometimes (ref never)	0.4 (0.19, 1.07)	0.11 (0.04, 0.33)***	1.4 (0.52, 4.00)	4.8 (1.72, 13.85)**	0.6 (0.34, 1.39)	0.5 (0.24, 1.27)	0.6 (0.30, 1.52)	1.0 (0.48, 2.28)	1.3 (0.70, 2.66)	2.5 (1.14, 5.80)*

*p < 0.05, **p < 0.01, ***p < 0.001

**Table 5:** Adjusted odds ratios (and 95 % confidence intervals) from multiple logistic regression analysis of associations between program exposure and couple communication, justification of IPV, any form of IPV, and physical, psychological, and verbal violence at end line (*n* = 435) and long-term follow-up (LTFU) (*n* = 399)

Variable	Couple communication		Justification of IPV		Any form of IPV		Physical		Psychological		Verbal	
	End line	LTFU	End line	LTFU	End line	LTFU	End line	LTFU	End line	LTFU	End line	LTFU
Attended at least one individual and one group mentoring session (ref did not attend)	2.4 (1.54, 3.77)***	2.4 (1.56, 3.81)***	0.63 (0.40, 0.98)*	0.50 (0.31, 0.83)**	0.48 (0.31, 0.76)**	0.48 (0.31, 0.77)**	0.71 (0.46, 1.11)	0.76 (0.38, 1.35)	0.55 (0.35, 0.86)**	0.42 (0.24, 0.74)***	0.51 (0.33, 0.79)**	0.56 (0.35, 0.90)**
Employment (ref farming) Employer/self-employed	1.5 (0.66, 3.64)	0.7 (0.37, 1.69)	1.1 (0.52, 2.35)	0.2 (0.95, 0.90)*	0.7 (0.35, 1.49)	2.0 (0.96, 4.32)	0.8 (0.40, 1.93)	1.2 (0.43, 3.48)	1.2 (0.58, 2.63)	2.4 (1.05, 5.54)*	0.5 (0.35, 0.901)	0.72 (0.46, 1.12)
Partial/full payment of bride price (ref no payment)	1.3 (0.82, 2.09)	0.78 (0.47, 1.68)	0.69 (0.44, 1.09)	0.50 (0.29, 0.86)**	1.2 (0.79, 1.87)	1.1 (0.66, 1.85)	1.2 (0.79, 2.01)	0.9 (0.44, 1.85)	0.97 (0.61, 1.54)	0.67 (0.36, 1.24)	1.2 (0.81, 1.95)	1.1 (0.67, 1.87)
Couple communication (ref none)	Not included	Not included	0.6 (0.38, 1.88)	0.6 (0.39, 1.09)	0.5 (0.32, 0.88)*	0.9 (0.61, 1.60)	0.4 (0.30, 0.78)**	0.6 (0.32, 1.77)	0.8 (0.49, 1.31)	1.0 (0.58, 1.90)	0.4 (0.28, 0.74)	0.8 (0.48, 1.30)
Alcohol use, past month (ref never/no drinking) 2 days or less More than 2 days	0.7 (0.45, 1.29) 0.5 (0.29, 0.92)*	1.4 (0.80, 2.48) 0.4 (0.29, 0.83)**	1.1 (0.70, 1.88) 1.4 (0.85, 2.60)	1.2 (0.67, 2.21) 2.3 (1.32, 4.22)**	1.9 (1.19, 3.08)** 1.4 (0.84, 2.55)	1.0 (0.62, 1.81) 3.1 (1.82, 5.34)***	1.2 (0.77, 2.06) 1.6 (0.93, 2.87)	1.5 (0.65, 3.60) 4.0 (1.89, 8.51)***	1.9 (1.26, 4.02) 1.6 (0.93, 2.98)	2.0 (1.01, 4.20)* 3.2 (1.67, 6.47)***	1.7 (1.12, 2.84)* 1.4 (0.84, 2.51)	0.8 (0.47, 1.51) 2.9 (1.70, 5.04)***
Threatened with physical violence often/sometimes (ref never)	1.7 (1.00, 2.86)*	0.8 (0.47, 1.68)	1.7 (1.01, 3.05)*	1.0 (0.54, 2.08)	2.4 (1.48, 4.09)***	0.7 (0.42, 1.43)	1.6 (0.84, 2.82)	0.7 (0.34, 1.75)	2.2 (1.26, 4.02)**	1.4 (0.64, 3.17)	1.8 (1.12, 2.84)*	0.6 (0.33, 1.18)
Physical punishment often/sometimes (ref never)	0.8 (0.42, 1.83)	0.73 (0.32, 1.69)	1.5 (0.71, 3.40)	4.6 (1.44, 15.0)**	1.8 (0.90, 3.57)	4.5 (1.83, 11.44)***	2.4 (1.02, 5.89)*	1.7 (0.55, 5.74)	1.3 (0.60, 3.05)	4.8 (1.00, 23.2)*	2.4 (1.16, 5.00)*	5.3 (1.97, 14.2)***

*p < 0.05, **p < 0.01, ***p < 0.001

At longer term follow-up, no significant differences were observed in background characteristics between exposed and unexposed men (Table 2). The one exception was the experience of physical violence as a child. This occurred significantly more frequently among unexposed versus exposed men (*p* value <0.05).

Table 3 presents levels of key parenting and partner relationship outcomes across baseline, end line, and long-term follow-up. Overall, there were decreases in perpetration of physical punishment and all forms of IPV, attitudes rejecting both forms of violence, and increases in positive parenting and confidence in using nonviolent discipline over time.

The intervention had positive effects on parent-child interaction, positive parenting practices, and attitudes and practices related to using physical punishment (Table 4). Men exposed to the intervention had significantly greater odds of practicing positive parenting, such as rewarding or praising the child for good behavior, showing the child affection, or taking the child some place special, at end line (aOR 1.6, CI 1.01, 1.63, *p* < 0.05) but not over the longer term (aOR 1.1, CI 0.65, 1.96).

At end line, men who attended at least one individual and one group mentoring session had twice the odds as men who did not to spend time with their child in activities such as playing, singing songs, naming, and counting things (aOR 2.1, CI 1.05, 4.33, *p* < 0.01). This positive relationship was observed at longer term follow-up with participating men having increased odds of more parent-child interaction (aOR 2.9, CI 1.48, 6.01, *p* < 0.01). Men who participated in mentoring sessions as compared to men who did not were more likely to disagree with statements about the use of physical child punishment such as, “Parents should teach a child who misbehaves by spanking or hitting him/her” at end line (aOR 1.6, CI 1.09, 2.49, *p* < 0.01) and at longer term follow-up (aOR 2.2 CI 1.43, 3.47, *p* < 0.001). Exposed men had significantly greater odds of feeling confident to manage their child’s behavior without resorting to use of physical violence or threats of violence in the short term (aOR 2.5, CI 1.50, 4.28; *p* < 0.001) and over the longer term (aOR 2.4; CI 1.55, 3.98, *p* < 0.001). Exposure to the intervention was associated with lower odds of using physical punishment at longer term follow-up (aOR 0.52, CI 0.32, 0.82, *p* < 0.01), but not in the short term.

Results of multiple logistic regression analyses testing the association between participation in the intervention and key partner relationship outcomes are presented in Table 5. Men who participated in individual and group mentoring sessions had twice the odds as men who did not tell their partner that they appreciated them, listen to their partner, and discuss with their partner things that make them happy or frustrated in both the short term (aOR 2.4, CI 1.54 3.77, *p* < 0.001) and at longer term follow-up (aOR 2.4, CI 1.56, 3.89, *p* < 0.001). Exposed men as compared to unexposed men had significantly lower odds of justifying IPV for any reason at end line (aOR 0.63, CI 0.40, 0.98, *p* < 0.05) and at long-term follow-up (aOR 0.50, CI 0.31, 0.83, *p* < 0.01). This is particularly notable given the relatively light nature of the intervention, implemented by volunteers with fewer than 10 days of training over a 6-month period. Men who participated in any mentoring sessions versus those who did not were less likely to use any form of IPV at end line (aOR 0.48, CI 0.31, 0.76, *p* < 0.001) and at long-term follow-up (aOR 0.48, CI 0.31, 0.77, *p* < 0.001). Program participation was not associated with significant increases in rejection of traditional gender norms at end line (not shown).

## Discussion

These results show overall significant, positive effects of the intervention on increasing positive parenting practices, confidence in using nonviolent discipline, and lowering the odds of use of physical punishment, and use of psychological and verbal IPV. Physical violence declined over time among the entire sample from 38 % at baseline to 12 % at long-term follow-up. Exposure to the intervention was significantly associated with attitudes that reject use of physical punishment and IPV. Men exposed to mentoring sessions had almost twice the odds of using positive parenting than unexposed men. The intervention had significant positive effects on couple communication but more limited effects on attitudes justifying IPV and no effect on gender norms. There was a significant effect of the intervention on use of physical punishment at long-term follow-up (aOR 0.84 CI 0.55, 0.82, *p* value < 0.01) but not at end line. Alcohol use for more than 2 days in the past month was significantly associated with perpetration of all forms of IPV and with attitudes supportive of using physical punishment. As noted in the literature, exposure to violence as a child was significantly associated with use of physical punishment and IPV.

Other studies of parenting interventions in post-conflict settings aimed at reducing CM through parent education had similar results in Liberia (Sim et al. 2014b) and Thailand (Sim et al. 2014a). Emerging evidence suggests that IPV programs could have positive effects on parenting (Kyegombe et al. 2015). The significant effects of REAL Fathers on couple communication are promising. As Doyle et al. (2014) have noted, couple communication may be crucial in negotiating or agreeing on changes in roles in the household as fathers take on more housework or child care responsibilities that have traditionally been for women. Based on results of increased couple communication and the significant association with physical violence, couple communication skills could be an important determinant of IPV and CM.

The limited effects on men’s agreement with traditional gender roles at end line demonstrate the challenge in addressing the underlying norms for men and women in the family context, particularly, in a short-term intervention. Additional engagement with wives or other influential individuals in the lives of new parents could contribute to more significant change in attitudes and norms related to gender roles. While engaging men in reflecting on roles and expectations of women and men as parents is an important aspect of the REAL Fathers intervention, sustained change in gender roles likely requires more long-term support and broader community involvement. As many structural interventions such as needle exchange programs in HIV prevention have shown, behavior change can precede shifts in norms. In this case, the behavior changes in practicing nonviolence in parenting and intimate partner relationships could contribute to changes in attitudes and expectations related to gender roles after the new practices have been adapted. Analysis of changes in agreement with traditional gender norms over the longer term is ongoing.

Limitations in this study should be mentioned. First, cause and effect relationships cannot be determined from cross-sectional data. Panel data would have provided greater statistical power to detect differences in exposed versus unexposed men, and contributions of group versus individual mentoring to outcomes could have been assessed. Unique identification codes are used to protect the confidentiality of study participants by using codes rather than names to identify study participants. Determining a unique identification code is a challenge in contexts where birth dates and other personally identifying information may not be reliable. This presents the need to link the participant’s name to their unique identification code, which is a concern for maintaining confidentiality. In addition, due to concerns related to relatively high rates of IPV in this setting, and few referral resources for responding to cases of IPV, wives and partners of the young fathers were not surveyed; therefore, these results rely on self-reported use of either form of violence. Limitations in validating self-reported data is a common problem in IPV research. Using other sources of available data in an effort to triangulate findings may be possible. In the case of Uganda, there is a sub-county and district-level gender-based violence (GBV) reporting system in the region. The reporting systems likely include only a portion of the actual cases, however, because some cases may be reported only to the traditional leaders and not through the governmental actors in the referral system or are not reported at all.

These limitations aside, the results suggest that addressing physical punishment and IPV in a single intervention can lead to significant reductions in the use of physical punishment over the longer term and in IPV over both short and longer term. These results contribute to the growing interest in interventions that address the double jeopardy of CM and IPV and show that targeting young fathers can be effective. Testing the integration of these combined, relatively simple parenting approaches across sectors including economic strengthening programs, maternal and child health, and reproductive and sexual health would be a major contribution to ongoing efforts in violence prevention.
